# Genome of *Laudakia sacra* Provides New Insights into High-Altitude Adaptation of Ectotherms

**DOI:** 10.3390/ijms231710081

**Published:** 2022-09-03

**Authors:** Chaochao Yan, Zhi-Yi Zhang, Yunyun Lv, Zeng Wang, Ke Jiang, Jia-Tang Li

**Affiliations:** 1 CAS Key Laboratory of Mountain Ecological Restoration and Bioresource Utilization & Ecological Restoration and Biodiversity Conservation Key Laboratory of Sichuan Province, Chengdu Institute of Biology, Chinese Academy of Sciences, Chengdu 610041, China; 2 College of Life Science, Neijiang Normal University, Neijiang 641100, China; 3 University of Chinese Academy of Sciences, Beijing 101408, China; 4 Center for Excellence in Animal Evolution and Genetics, Chinese Academy of Sciences, Kunming 650223, China; 5 Mangkang Biodiversity and Ecological Station, Tibet Ecological Safety Monitor Network, Changdu 854500, China

**Keywords:** lizard, de novo genome, comparative genomics, hypoxia, UV damage

## Abstract

Anan’s rock agama (*Laudakia sacra*) is a lizard species endemic to the harsh high-altitude environment of the Qinghai–Tibet Plateau, a region characterized by low oxygen tension and high ultraviolet (UV) radiation. To better understand the genetic mechanisms underlying highland adaptation of ectotherms, we assembled a 1.80-Gb *L. sacra* genome, which contained 284 contigs with an N50 of 20.19 Mb and a BUSCO score of 93.54%. Comparative genomic analysis indicated that mutations in certain genes, including *HIF1A*, *TIE2*, and NFAT family members and genes in the respiratory chain, may be common adaptations to hypoxia among high-altitude animals. Compared with lowland reptiles, *MLIP* showed a convergent mutation in *L. sacra* and the Tibetan hot-spring snake (*Thermophis baileyi*), which may affect their hypoxia adaptation. In *L. sacra*, several genes related to cardiovascular remodeling, erythropoiesis, oxidative phosphorylation, and DNA repair may also be tailored for adaptation to UV radiation and hypoxia. Of note, *ERCC6* and *MSH2*, two genes associated with adaptation to UV radiation in *T. baileyi*, exhibited *L. sacra*-specific mutations that may affect peptide function. Thus, this study provides new insights into the potential mechanisms underpinning high-altitude adaptation in ectotherms and reveals certain genetic generalities for animals’ survival on the plateau.

## 1. Introduction

As the highest plateau on Earth, the Qinghai–Tibet Plateau is characterized by extreme environments, including low partial pressure of oxygen (PaO_2_) and high ultraviolet (UV) radiation. Maintaining oxygen homeostasis is vital to the survival of all vertebrate species, and organisms initiate several responses to alleviate hypoxic stress [1]. For example, mitochondria respiration stimulates the cellular accumulation of reactive oxygen species (ROS) in a low oxygen environment, which can cause damage to lipids, proteins, DNA, and ultimately lead to cell death [2,3]. HIF1A, a key modulator of the cellular response to hypoxic stress, will be stabilized and recruited into mitochondria to reduce ROS through the inhibition of respiratory chain activity [3,4]. Other cellular and tissue reactions to hypoxic challenge include expanded cardiovascular remodeling to increase ventilation and cardiac output, and increased red blood production to enhance O_2_ transport [1]. Similar to hypoxia, high UV radiation also threatens the survival of living beings as UV-induced DNA lesions can cause mutations and death if unrepaired [5]. Properly functioning DNA repair systems (e.g., base excision repair, nucleotide excision repair, recombinational repair, and so on) are thus essential, and they evolved to be more efficient in organisms exposed frequently to high UV radiation [6,7].

To understand the molecular mechanisms underpinning the survival of high-altitude dwelling lives, many recent studies have explored the adaptive evolution of animals on the Qinghai–Tibet Plateau, including mammals [6,8], reptiles [7,9], birds [10,11], and amphibians [12,13]. In reptiles, the Tibetan hot-spring snake’s (*Thermophis baileyi*) genetic adaptations were found for UV radiation, hypoxia, and thermal extremes based on genomic data [7,14]. Notably, several DNA repair genes, including *ERCC6*, *SMARCAL1*, *USP7*, *MEIOB*, *DMAP1*, and *MSH2*, show positive selection in *T. baileyi* and are related to the p53 pathway, which is activated under cellular stress caused by external stimuli, including hypoxia and UV radiation. Furthermore, *EPAS1* evolved adaptively in *T. baileyi* as well as Tibetan mammalian species to balance erythrocyte levels under hypoxic stress. However, a study comparing the high altitude-dwelling Sagus Kul lizard (*Phrynocephalus erythrurus*) and its lowland-dwelling relative *Phrynocephalus putjatia* using transcriptome data has indicated that *AGTRAP* and *UBE2D1* are positively selected for coping with hypoxia, while *RFC1*, *DSCC1*, *LIG4*, *XRCC3*, *XRCC4*, *NBN*, *FAM175A*, *SMC5*, *MMS22L*, and *YY1* evolved adaptively in response to UV damage [9]. Therefore, whether convergence in adaptation to high altitude occurred between snakes and lizards, or whether different strategies were adopted remains unknown.

**Figure 1:**
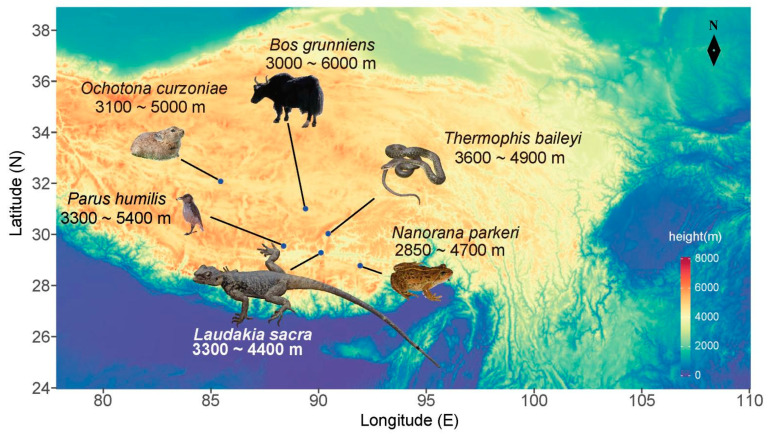
Plateau species included in the study, with habitat elevation listed below each species.

Anan’s rock agama (*Laudakia sacra*) is an endemic Tibetan lizard species within the family Agamidae. It is primarily distributed around the Yarlung Zangbo River and its tributary river valley, extending west to Lazi County and east to Milin County [15]. Its preferred natural habitats include rocky areas and freshwater wetlands at altitudes of 3300–4400 m (Figure 1) [16]. Thus, this lizard species is an ideal model system to further study the genetic basis of local adaptation to extreme conditions in ectothermic animals.

Here, we assembled an *L. sacra* genome using next-generation sequencing (NGS) and third-generation sequencing (TGS) data. Furthermore, to explore the potential genetic basis for high-altitude living and possible convergence in genetic mechanisms, we compared the *L. sacra* genome to that of 49 species from various classes, including five species native to the Qinghai–Tibet Plateau that belong to Amphibia (*Nanorana parkeri*), Reptilia (*T. baileyi*), Aves (*Parus humilis*), and Mammalia (*Ochotona curzoniae* and *Bos grunniens*) (Figure 1). The differences and convergence between snakes (i.e., *T. baileyi*) and lizards (i.e., *L. sacra* and *P. erythrurus*) for high-altitude adaptation were also investigated in the current study.

## 2. Results

### 2.1. De Novo Assembly of L. sacra Genome

**Figure 2:**
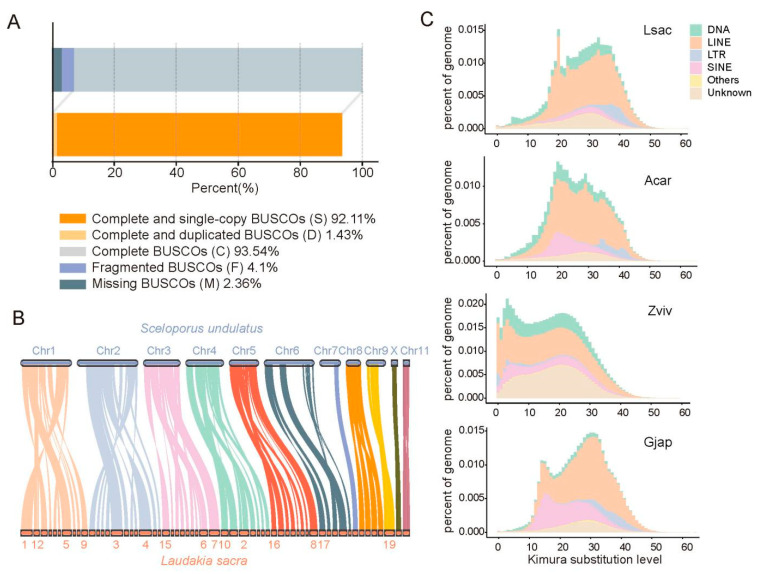
Assessment of *Laudakia sacra* genome assembly and comparison with other lizard genomes. (**A**) BUSCO assessment of *L. sacra* assembly. (**B**) Genome-wide synteny between *L. sacra* and *Sceloporus undulatus*. (**C**) Transposable element (TE) accumulation history in different lizards. The X-axis is the CpG-adjusted Kimura distance from consensus in TE library. The Y-axis is the percentage of TE occupancy in genome. Copies clustered on right of graph markedly diverged from their corresponding consensus, potentially corresponding to ancient copies, while sequences on the left may correspond to recent copies. Lsac, *L. sacra*; Acar, *Anolis carolinensis*; Zviv, *Zootoca vivipara*; Gjap, *Gekko japonicus*.

In total, 143.60 Gb of clean Illumina paired-end reads and 162.90 Gb of PacBio Sequel II subreads was generated from a male *L. sacra* individual (Table S1), representing 84.47- and 95.82-fold coverage of the estimated 1.70 Gb genome, respectively. The PacBio reads were used for initial assembly, which was then polished with short paired-end reads to generate an assembly containing 284 contigs with an N50 of 20.19 Mb and a total length of 1.80 Gb (Table S2). Approximately 90% of the assembly was contained in the 89 longest contigs, with the largest spanning 77.01 Mb (Table S2). In total, 93.54% of complete vertebrate BUSCO genes, including 92.11% of single-copy genes, were captured in the *L. sacra* assembly (Figure 2A; Table S3). The alignment of this assembly with the high-quality *Sceloporus undulatus* (Squamata: Phrynosomatidae) reference genome exhibited a strong syntenic relationship (Figure 2B) [17]. The long contig N50, high BUSCO completeness score, and strong syntenic relationship to the high-quality *S. undulatus* reference genome indicated a relatively good assembly quality of the *L. sacra* genome.

In total, 19,893 protein-coding genes were predicted, 97.43% of which could be annotated in public databases (Table S4), and the length distribution of the genes, introns, and CDs of the gene-set are similar to typical published lizard and snake genomes (Figure S1). Repetitive sequences accounted for 42.25% of the genome, consisting of 0.55% tandem repeats (e.g., simple sequence repeats (SSRs) and simple tandem repeats (STRs)) and 36.78% transposable elements (TEs) (Table S5). In the *L. sacra*, *A. carolinensis*, *G. japonicus*, and *Z. vivipara* genomes, TEs with high divergence (Kimura distance ≥ 20) from their corresponding consensus exhibited high genome coverage, indicating relatively ancient bursts of TEs (Figure 2C). This TE bursts pattern also reflected the tendency of changes in the effective population size (*Ne*) of *L. sacra* through time (Figure S2). In comparison, the TEs in the *Z. vivipara* genome also exhibited a recent burst, and unknown TEs expanded more actively in the genome compared to that in other lizards (Figure 2C).

### 2.2. Phylogenetic Reconstruction

**Figure 3:**
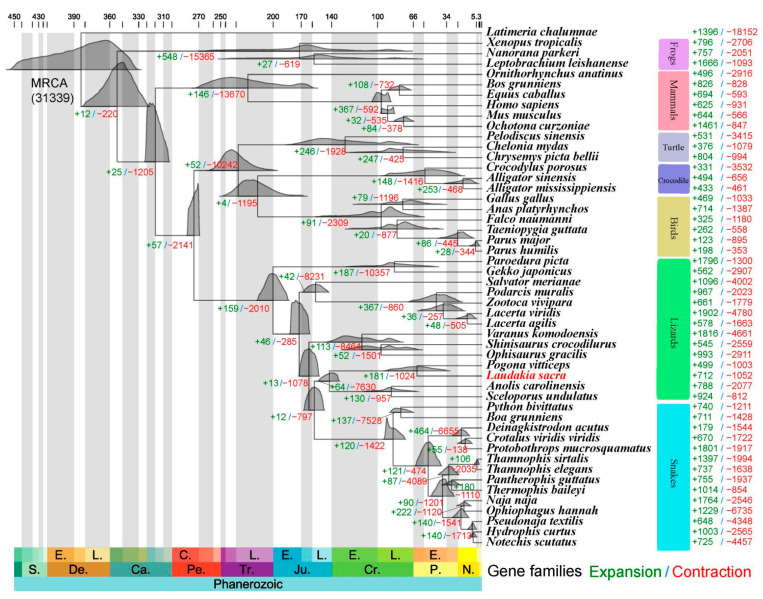
Reconstructed phylogeny of 50 species using single-copy orthologous groups identified in their genomes. Phylogenetic tree is scaled by divergence time, with geological times indicated below and divergence times in Mya provided above. *Laudakia sacra* whose genome is sequenced in this study is highlighted in red. Green and red numbers indicate number of significantly expanded (+) and contracted (−) gene families, respectively. E., early; L., late; C., Cisuralian; S., Silurian; De., Devonian; Ca., Carboniferous; Pe., Permian; Tr., Triassic; Ju., Jurassic; Cr., Cretaceous; P., Paleogene; N. Neogene.

With 374 single-copy orthologous groups identified across the genomes of *L. sacra* and 49 other species (Table S6), we constructed a maximum-likelihood tree, including time calibrations based on fossil records or inferred divergence time (Figure 3) [18]. Congruent with previous studies, Markov chain Monte Carlo (MCMC) analysis indicated that divergence between Lepidosauria (snakes and lizards) and Archosauromorpha (turtles, crocodilians, and birds) occurred around 270.0 million years ago (Mya) during the Permian period [7], and the emergence of the common ancestor of Agamidae, to which *Laudakia* and *Pogona* belong, was no earlier than the Late Cretaceous (Figure 3) [19].

Based on the reconstructed phylogeny, OrthoFinder defined 31,340 orthologous groups using genomic data of the 50 studied species, with 181 of these groups exhibiting significant expansion in the *L. sacra* genome (Figure 3). These expanded gene families were significantly (*p*-value < 0.01) enriched in several Kyoto Encyclopedia of Genes and Genomes (KEGG) pathways, including the parathyroid hormone synthesis and nucleotide-binding oligomerization domain (NOD)-like receptor signaling pathway (Table S7). In addition, based on Gene Ontology (GO) analysis, the expanded gene families were significantly (adjusted *p*-value < 0.05) enriched in biological processes such as DNA repair, the regulation of ATPase activity, calcium ion transport, vasodilation, and heart growth (Figure S3; Table S8).

### 2.3. Positively Selected Genes (PSGs) and Quickly Evolving Genes (QEGs) Analyses

**Figure 4:**
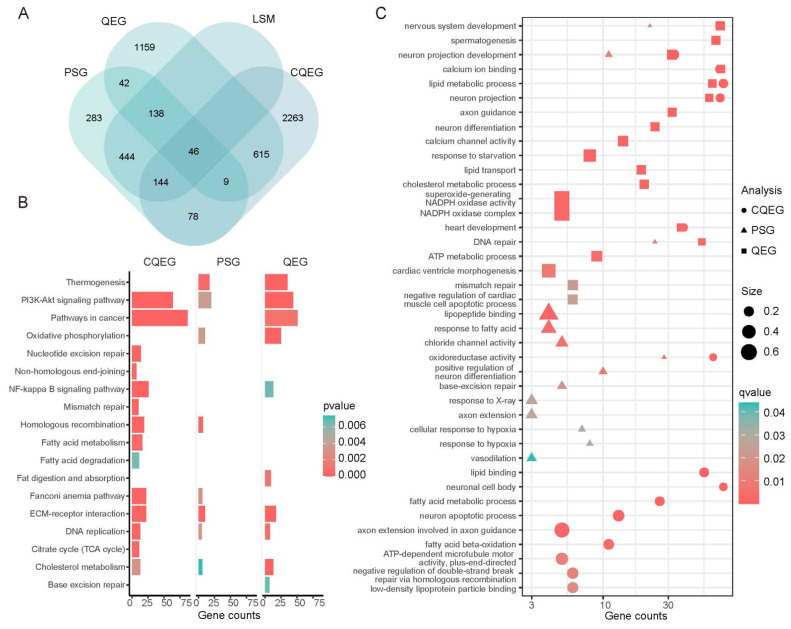
Comparative genomic analyses and functional enrichment of target genes. (**A**) Venn diagram showing numbers of target genes in different categories. PSGs and QEGs are positively selected genes and quickly evolving genes in *L. sacra* genome, respectively. CQEG represents common QEG. LSM represents genes with *L. sacra*-specific mutations. (**B**) Significantly enriched KEGG pathways in different gene categories. Color indicates enrichment *p*-value. Length of bar represents number of genes in a certain gene category. (**C**) Significantly enriched GO terms in different gene categories are represented by different shapes. Shape size represents number of genes in a certain category divided by the number of annotations for the corresponding GO term. Color indicates adjusted enrichment *p*-value (i.e., q-value).

We analyzed single-copy orthologs shared among the *L. sacra* and 29 lowland animal genomes, setting *L. sacra* as the foreground. In the *L. sacra* genome, we identified 2009 QEGs (i.e., genes with high rates of molecular evolution) and 1184 PSGs, including 772 showing *L. sacra*-specific mutations (LSM) (Figure 4A; Table S9). We further analyzed the single-copy orthologous groups shared by all 50 species and identified 3155 common QEGs (CQEGs) with signals of convergent acceleration in gene-wide rates in species endemic to the Qinghai–Tibet Plateau, including *B. grunniens*, *L. sacra*, *N. parkeri*, *O. curzoniae*, *P. humilis*, and *T. baileyi* (Figure 4A).

The PSGs and QEGs in the *L. sacra* genome were enriched in pathways activated during the cellular response to hypoxia (e.g., PI3K-Akt signaling pathway and extracellular matrix (ECM)-receptor interactions) and were also involved in DNA repair (e.g., Fanconi anemia pathway and base excision repair) (Figure 4B,C; Tables S10–S13) [20,21,22,23]. Cardiac ventricle morphogenesis and metabolic processes, such as thermogenesis, oxidative phosphorylation, and cholesterol metabolism, were also significant categories (Figure 4B,C; Tables S10–S13). PSGs with *L. sacra*-specific mutations were significantly enriched in various functions, including the response to X-ray, the response to hypoxia, and DNA repair (Figure 4C; Table S10).

Similarly, common QEGs in plateau species were enriched in categories related to cellular stress response, DNA repair, calcium transport, energy metabolism, and the regulation of the cardiovascular system (Figure 4B,C; Tables S14 and S15).

### 2.4. Potential Genetic Basis of Adaptation to Plateau Environments

**Figure 5:**
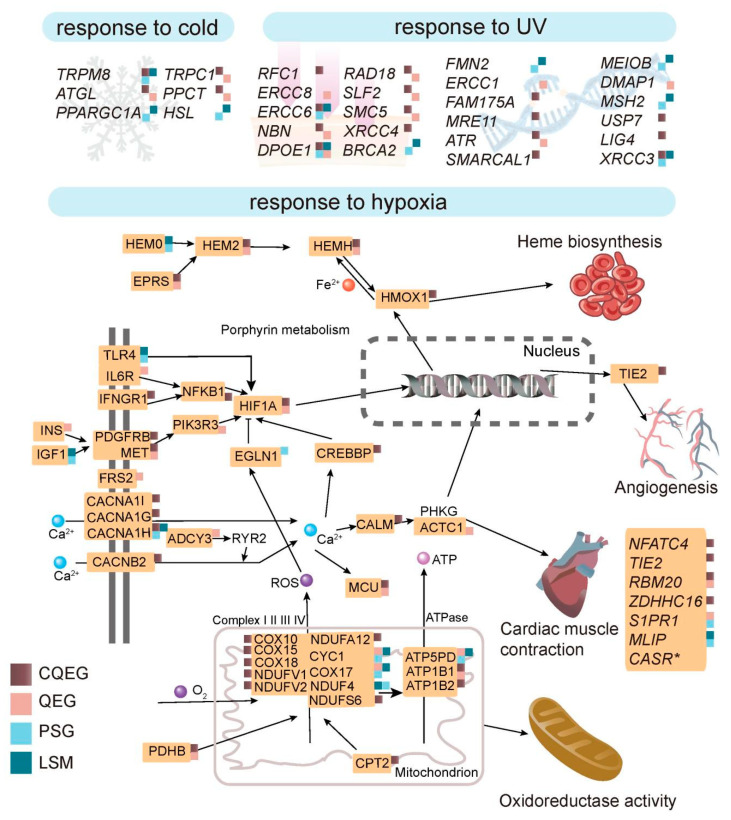
Genes and pathways associated with plateau animal adaptation to hypoxia, cold temperature, and high UV radiation. Hypoxia-activated pathways that regulate erythropoiesis, cardiovascular development, and oxidative phosphorylation in plateau animals are depicted in detail. Different categories of genes, i.e., CQEGs, QEGs, PSGs, and genes with *L. sacra*-specific mutations (LSM) are indicated by squares with different colors. Expanded *CASR* in *L. sacra* is marked with an “*”.

In the *L. sacra* genome, several genes (e.g., *TRPM8*, *ATGL*, *HSL*, and *PPARGC1A*) that play a role in thermoregulation through lipid metabolism were either positively selected or evolved with high rates of molecular evolution (Figure 5; Table S16) [24,25,26,27,28,29]. Among them, *TRPM8*, *ATGL*, *TRPC1*, and *PPCT* were identified as common QEGs (Figure 5).

**Figure 6:**
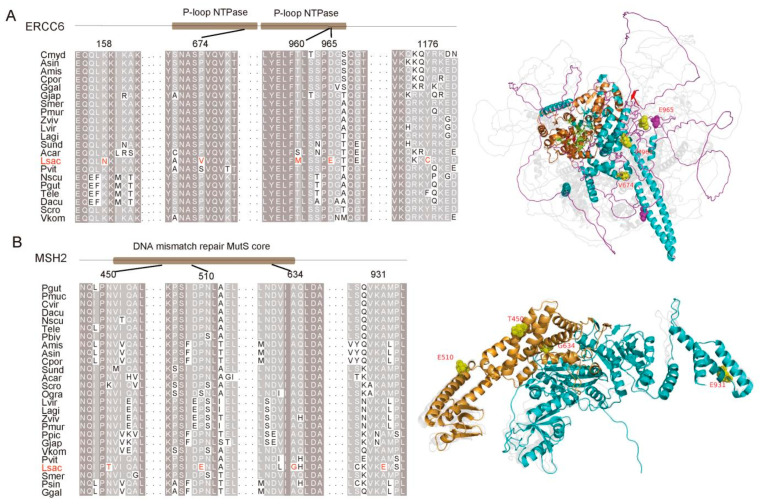
Sequence alignment of ERCC6 and MSH2. (**A**) Sequence alignment showing conserved replacements (red) on *L. sacra* ERCC6. Positions of mutations on P-loop NTPase domain are indicated. Superimposed protein tertiary structures on the right show mutations on *L. sacra* ERCC6. (**B**) Sequence alignment showing N450T, P510E, A634G, and A931E mutations on *L. sacra* MSH2. Mutations in the functional domain are represented by yellow spheres. Full names of the species’ name abbreviations are provided in Table S6.

In addition, many genes important for maintaining normal DNA structure exhibited convergently accelerated gene-wide rates in the high-altitude animals, including *USP7*, *XRCC3*, *MRE11*, *ERCC6*, *SMARCAL1*, *RFC1*, *LIG4*, and so on (Figure 5; Table S16) [9,30,31,32,33,34]. In contrast, DNA repair genes such as *ERCC1*, *ERCC8*, and *DMAP1* evolved quickly in *L. sacra* [35,36], while *BRCA2*, *FMN2*, *MSH2*, *ERCC6*, *MEIOB*, and *DPOE1* were positively selected and showed *L. sacra*-specific mutations (Figure 5) [22,32,37,38,39,40]. Among the PSGs in the *L. sacra* genome, *MSH2* and *ERCC6*, which are associated with Tibetan hot-spring snake adaptation to UV radiation [7], displayed functional domain mutations (PROVEAN scores < −2.5). Three *L. sacra*-specific amino acid replacements on ERCC6 were found near the alpha-helices in the P-loop containing the nucleoside triphosphate hydrolase (P-loop NTPase) domain, responsible for nucleoside triphosphate hydrolysis [41], and the D965E substitution may have functional implications according to PROVEAN scores (Figure 6A). On MSH2, the *L. sacra*-specific amino acid replacements were found in the alpha-helices of the functional domain, one of which (i.e., A634G) was predicted to affect peptide function as its PROVEAN score was smaller than −2.5 (Figure 6B).

When facing a hypoxic challenge, HIF1A will be stabilized and modulate the responses of the circulatory system and mitochondria to low oxygen [2,3,42,43,44,45]. Our results showed that this master transcriptional regulator in hypoxia response pathway is a common QEG with signals of convergent acceleration in gene-wide rates in plateau animals (Figure 5).

We also found that various genes known to play a role in the proper functioning of the cardiovascular system evolved differently in the plateau animals compared with lowland wildlife. For example, *NFATC3*, *NFATC4*, *TIE2*, *RBM20*, and *ZDHHC16*, which regulate cardiac development and/or blood vessel morphogenesis [46,47,48,49], were identified as common QEGs (Figure 5; Table S16). *CASR*, which affects blood vessel morphology [50], was significantly expanded in the *L. sacra* genome (Figure 5). In addition, *HEM0* (*ALAS2*) and *MLIP*, which regulate erythropoiesis and cardiac homeostasis [51,52], respectively, showed positive selection in *L. sacra* (Figure 5). Of note, when compared with lowland reptiles, the functional domain of MLIP showed a mutation shared among the *L. sacra* and *T. baileyi*. The serine-to-proline replacement reversed the affinity of this site for water and was predicted to influence peptide function according to PROVEAN scores (Figure S4).

In the plateau animals, several genes essential in the mitochondrial electron transport chain (e.g., *COX10*, *COX15*, *NDUFV1*, and *NDUFS6*) exhibited convergently accelerated gene-wide rates as well (Figure 5) [51,53,54,55]. ATP5PD, a subunit of mitochondrial ATP synthase that utilizes the proton gradient established by the electron transport chain to synthesize ATP [56], was positively selected and harbored *L. sacra*-specific mutations (Figure 5).

## 3. Discussion

High-altitude adaptation studies of animals on the Qinghai–Tibet Plateau have primarily been conducted in mammals and birds, with relatively few studies on local adaptation in ectotherms. By completing the *L. sacra* reference genome, we provide the second assembly of reptiles endemic to the Qinghai–Tibet Plateau, which should facilitate our understanding of the genetic mechanism behind high-altitude adaptation in ectotherms. Together with the genomic data from 49 other animals, including five highland species, we also explored the general genetic basis for surviving on the plateau and found several genes in the molecular pathways crucial for coping with high-altitude stress (i.e., cardiovascular morphogenesis, oxidative phosphorylation, thermogenesis, and DNA repair) positively selected and/or quickly evolved in plateau animals [7,8,9,57].

### 3.1. Adaptation to Hypoxia

Insufficient oxygen availability is a precursor to several cardiovascular diseases, such as atherosclerosis, pulmonary hypertension, and heart failure. NFATC3 and NFATC4 are two Ca^2+^-regulated transcription factors that transactivate genes involved in cardiac morphogenesis, vasculature development, and vascular smooth muscle contractility [58,59]. The activation of *NFATC3* and *NFATC4* by hypoxia is associated with pulmonary hypertension due to the thickening and stiffening of pulmonary arteries [58,59]. *TIE2* encodes a vascular endothelium-enriched receptor important for angiogenesis, which plays a role in reducing hypoxia and tumor growth [43]. In the current study, these genes were identified as common QEGs (Figure 5; Table S16), and their mutations in high-altitude animals may be necessary for counteracting the stress exerted by low-oxygen environments on the cardiovascular system.

Our results also showed that *CASR* was expanded in the *L. sacra* genome (Figure 5; Table S16). The calcium-sensing receptor (CaSR) plays a key role in activating NLRP3-mediated signaling cascades (e.g., NOD-like receptor signaling pathway) related to the occurrence of cardiovascular diseases and ischemic brain injury under hypoxic conditions [60,61,62]. It maintains calcium homeostasis and has protective effects on the peripheral vascular system, including vasorelaxation, elasticity maintenance, and anti-proliferation [50]. However, CaSR can also bind to a hypoxia-induced mitogenic factor (HIMF), resulting in pulmonary vascular remodeling and pulmonary hypertension [63]. The expansion of *CASR* in *L. sacra* may be to maintain normal peripheral vascular system function or to retain surplus CaSRs after HIMF binding to maintain normal pulmonary artery morphology. Similarly, MLIP regulates cardiac homeostasis to prevent hypertrophic cardiomyopathy and possibly pulmonary hypertension, two diseases that can result from hypoxia [51,64]. *MLIP* was positively selected in *L. sacra* and contained a potential functional mutation shared with *T. baileyi* (Figure 5; Figure S4; Table S16), suggesting its importance in cardiac adaptation to harmful stimuli in highland reptiles. Mutations in the positively selected gene *HEM0* in *L. sacra* may also be an adaptation to low-oxygen environments (Figure 5; Table S16), as the upregulation of *HEM0* during hypoxia is thought to promote erythropoiesis for oxygen-carrying [65]. Increasing blood cells is an adaptation found in Tibetan chickens, although it may have evolved via a different molecular mechanism [11].

Mitochondria burn oxygen and generate energy for the body through redox reactions along the electron transport chain, which consists of four enzymes (complexes I to IV). Inefficient electron flux between the complexes during hypoxia can result in lower ATP production, which reduces the cellular respiration rate to prevent the accumulation of detrimental ROS [4,66]. NADH dehydrogenase family members (e.g., *NDUFA12*, *NDUFS6*, *NDUFV1*, and *NDUFV2*) and cytochrome c oxidase (COX) assembly factors (e.g., *COX10*, *COX15*, and *COX18*) are integral components of complexes I and IV, respectively. In our study, these genes showed convergently accelerated gene-wide rates in the upland animals (Figure 5; Table S16), indicating potentially important roles in mediating normal energy production and ROS accumulation under hypoxic challenge. The synthesis of ATP through mitochondrial oxidative phosphorylation is completed by ATP synthase, which consists of several subunits. Here, the ATP5PD subunit was positively selected in *L. sacra* (Figure 5; Table S16). As the loss-of-function of this subunit is related to ATP synthesis dysfunction and ROS accumulation [67], mutations in the peptide may be necessary for the species to regulate ATP production in low-oxygen environments.

Central to the cellular responses to hypoxia lies HIF1A [2]. This master transcriptional regulator can interact with several genes such as *TIE2*, *NFATC4*, and *HEM0* to help the cardiovascular system function adaptively under hypoxic conditions [42,44,65,68,69]. Furthermore, HIF1A is recruited to the mitochondria in response to ROS-induced oxidative stress and can attenuate apoptosis and ROS production by regulating the expression of the complex I and IV subunits (e.g., NDUFA4L2 and COX4, respectively) [3]. Unsurprisingly, as an important gene in hypoxia adaptation, *HIF1A* showed rapid evolution in the highland animals (Figure 5; Table S16).

Previous studies have hypothesized that indigenous plateau mammals rely on *NOS3* (a regulator of vascular morphology) and *PKLR* (a catalyzer of glycolysis) to cope with hypoxia [6]. *EPAS1* (a regulator of erythropoiesis) is also thought to have evolved convergently in *T. baileyi* and Tibetan mammals, possibly in response to the need for balanced erythrocyte levels under hypoxic stress [7]. However, after integrating additional genomes of plateau-endemic animals, we found that mutations in genes such as *HIF1A*, *TIE2*, and NFAT family members and those in the electron transport chain may be a more general adaptation to low-oxygen environments among high-altitude animals (Figure 5; Table S16).

### 3.2. Adaptation to UV Radiation

Several DNA repair genes, including *ERCC6*, *SMARCAL1*, *USP7*, *DMAP1*, and *MSH2*, are positively selected in Tibetan hot-spring snakes, likely in response to high levels of UV exposure [7]. According to our results, the adaptive evolution of *ERCC6*, *SMARCAL1*, and *USP7* was not restricted to the highland snake (Figure 5; Table S16). Similarly, positively selected DNA repair genes in the upland-dwelling Sagus Kul lizard (i.e., *RFC1*, *LIG4*, *XRCC3*, *XRCC4*, *NBN*, *FAM175A* and *SMC5*) may also have evolved adaptively in other plateau animals (Figure 5; Table S16) [9]. In *L. sacra*, *ERCC6* and *MSH2* showed positive selection, with species-specific mutations predicted to affect protein function (Figure 5 and Figure 6; Table S16). Moreover, *SMC5*, *NBN*, and *DMAP1* exhibited a higher rate of molecular evolution in *L. sacra* (Figure 5; Table S16). Thus, these five genes may have also been tailored to meet the needs of *L. sacra* to maintain normal DNA replication and transcription under high UV radiation.

### 3.3. Adaptation to Cold

Animals, especially ectothermic species, must develop cold tolerance mechanisms to adapt to the lower temperatures of high-altitude environments. Indeed, we found that *TRPM8* and *ATGL*, which are responsible for adipocyte thermogenesis, exhibited signals of convergent acceleration in gene-wide rates in highland species (Figure 5; Table S16). The influx of calcium ions into the cytoplasm is highly correlated with heat production [70]. The well-known cold sensor TRPM8 on human white adipocytes can increase cytoplasmic calcium ions and heat production [27]. In addition, ATGL catalyzes the initial step of triglyceride hydrolysis in adipocyte and non-adipocyte lipid droplets [28]. Its presence in white adipose tissue and heart tissue is crucial for providing fatty acids for energy combustion and for meeting increased demands on the cardiovascular system under cold conditions [28]. Thus, in plateau animals, these genes may have evolved to increase lipid metabolism.

## 4. Materials and Methods

### 4.1. Genome Sequencing and Assembly

A male *L. sacra* was collected in Tibet (Latitude: 90°12′25″ N; Longitude: 29°20′48″ E; Altitude: 3675 m) and processed in accordance with the guideline for the ethical review of animal welfare of the People’s Republic of China. All the animal experiments reported in this study were approved by the Animal Experiment Ethics Committee in Chengdu Institution of Biology, Chinese Academy of Sciences (project identification code: CIBDWLL2022035).

Whole genomic DNA was isolated from the animal’s muscle tissue (sample name: LJT_LAB2020323) and used to construct continuous long reads (CLR) DNA libraries with an insert size of about 30 kb. The libraries were then sequenced on the PacBio Sequel II system at Frasergen company (Wuhan, China) to generate about 162.90 Gb data. For de novo assembly, approximately 143.60 Gb short-reads (generated by the Illumina HiSeq 4000 platform at Novogene company, Tianjing, China) were assembled into initial contigs using Platanus v1.2.4 with the following optimized parameters: -k 33 -t 8 -m 500 [71]. The contigs were then aligned against the PacBio reads with DGB2OLC to generate consensus contigs [72]. Base errors in the contigs were then polished based on PacBio long reads and Illumina short-reads using NextPolish v1.0.4 [73]. BUSCO v5.1.310 was used to estimate the completeness of the genome assembly [74].

### 4.2. Gene Structural and Functional Annotations

Repeat elements, including simple repeat sequences, tandem repeat elements, and transposable elements (TE), were firstly annotated and masked in the reference genome by using a Tandem Repeats Finder and RepeatMasker v4.1.0 [75]. The divergence level between the individual TE copies versus their consensus sequences was estimated using RepeatMasker built-in scripts based on CpG-adjusted Kimura distance.

Three approaches, including ab initio prediction, homology search, and transcriptome-based prediction, were then used independently for gene prediction in a repeat-masked genome. In homology search, the homologous protein sequences of relative lizard genomes were downloaded from Ensembl and were aligned to the assembly to get the gene structure information using GeMoMa v1.6.1 [76,77]. For RNAseq-based gene prediction, we used STAR v2.7.3a to align the filtered transcriptome data of mixed *L. sacra* tissues (heart, muscle, and liver) to the reference genome [78]. Then, StringTie v1.3.4d and PASA v2.3.3 were applied to assemble the transcripts and to predict open reading frames (ORFs), respectively [79,80,81]. For the de novo prediction, RNA-seq reads were firstly de novo assembled by StringTie v1.3.4d and analyzed with PASA v2.3.3 to produce a training set, which was then used to train Augustus v3.3.1 iteratively for gene prediction [82]. Finally, EVidenceModeler (EVM) v1.1.1 was used to produce an integrated gene set [81].

For the functional annotations of the protein-coding genes, BLASTp v2.7.1 was used to align the annotated proteins against the NR (non-redundant protein sequences in NCBI), SwissProt, and RefSeq databases (E value < 1 × 10^−5^). The NR BlastP results were processed using Blast2GO v5.2.514 to retrieve associated Gene Ontology (GO) terms [83]. We also used the KOBAS v3.0.3 database to obtain GO terms and KO identifiers of each gene [84]. The motifs and domains of each annotated gene were predicted by InterProScan against ProDom, PRINTS, Pfam, Gene3D, CCD, SMART, PANTHER, PROSITE, and SUPERFAMILY.

### 4.3. Phylogenetic Tree Construction and Divergence Time Estimation

Single-copy gene families in *L. sacra* and 49 other species were identified using Orthofinder v2.5.4 [85]. Then, four-fold degenerate sites in the CDS alignments of each single-copy gene family were extracted with self-made scripts. All four-fold degenerate sites were concatenated to construct a super-gene for each species, which were used for phylogenomic analysis. A maximum-likelihood tree was constructed using IQ-TREE v 1.6.520 with parameters: -nt 10 -st DNA -bb 1000 -alrt 1000 [86]. The divergence time of each branch was calculated with MCMCTREE in PAML version 4.9i [87]. Six calibrated divergence events were set according to the TimeTree website resource [18]: (1) ancestors of *Homo sapiens* and *Mus musculus* separated range from 85 to 97 Mya; (2) ancestors of *Anolis carolinensis* and *Pogona vitticeps* separated range from 139 to 166 Mya; (3) ancestors of *Boa constrictor* and *Naja naja* separated range from 70 to 92 Mya; (4) ancestors of *A. carolinensis* and *Zootoca vivipara* separated range from 166 to 184 Mya; (5) ancestors of *A. carolinensis* and *Gallus gallus* separated range from 273 to 284 Mya; (6) ancestors of *A. carolinensis* and *H. sapiens* separated range from 294 to 323 Mya. The correlated rates clock and the JC69 model in MCMCTREE were used for the calculation. The MCMC process was run for 100,000 iterations, after a burn-in of 20,000 iterations.

### 4.4. Gene Family Expansion and Contraction

We used CAFE v4.2.1 to perform gene family expansion and contraction analyses based on the tree constructed above [88]. The expanded and contracted gene families on each branch of the tree were detected by comparing the cluster size of each branch with the maximum-likelihood cluster size of the ancestral node leading to that branch; a smaller ancestral node indicates gene family expansion, whereas a larger ancestral node indicates family contraction. The significant size variation for each gene family was estimated based on the overall *p*-value (family-wide *p*-value in CAFE v4.2.1 calculated through 10,000 Monte Carlo resampling). The exact *p*-value for each branch or node was calculated with the Viterbi method to identify lineage-specific, significantly (overall *p*-value < 0.01) varied gene families.

### 4.5. Common Quickly Evolving Genes (CQEGs) in Plateau Animals

Single copy gene families identified among 50 genomes were firstly used for finding genes with high rates of molecular evolution (common quickly evolving genes, CQEGs). To identify CQEGs, we used the branch model, with high-elevation species set as the foreground branches and others set as background branches. Sequences of each single copy gene cluster were translated using self-made python scripts. The protein sequences of each cluster were aligned using PRANK v.150803 and trimmed with trimAl v1.4 in automated1 mode [89,90]. The trimmed alignments were back-translated by using self-made scripts and used for the following analyses. The branch model of CODEML in PAML4.9 was used to find CQEGs, convergent signatures of acceleration in the gene-wide rates of molecular evolution in plateau animals (*B. grunniens*, *L. sacra*, *N. parkeri*, *O. curzoniae*, *P. humilis*, and *T. baileyi*). Plateau species were set as the foreground branches and others as background branches. The null model was that the ω of each branch was equal, while the alternative model allowed more than one ω across branches. The *p*-values were calculated based on the likelihood ratio test (LRT) for the two models. A *p*-value < 0.01 represents significant CQEGs.

### 4.6. Positively Selected Genes (PSGs) and Quickly Evolving Genes (QEGs) in L. sacra Genome

A subset of single-copy genes in *L. sacra* and 29 other lowland species was used to find PSGs and QEGs in the *L. sacra* genome (Table S6). The branch model of CODEML in PAML4.9 was used to find QEGs in *L. sacra*. The null model was that the ω of each branch was equal, while the alternative model allowed more than one ω across branches. The *p*-values were calculated based on the likelihood ratio test (LRT) for the two models. A *p*-value < 0.01 represents significant QEGs.

The branch-site model of CODEML in PAML v4.919 was used to test for potential PSGs, in which the null hypothesis was that the ω value of each site on each branch was fixed to 1, whereas the alternative hypothesis was that the ω values of particular sites on the foreground branch were not fixed. The *p*-values for the likelihood ratio test (LRT) were then determined following the CODEML processes. According to Bayes Empirical Bayes analysis, the PSGs were defined based on a corrected *p*-value < 0.05 and at least one positively selected site with a posterior probability >0.95. In both analyses, *L. sacra* was set as the foreground branch, and 29 lowland species were set as background branches.

### 4.7. Species-Specific Mutations and Alignment Visualization

The alignments of single-copy genes were also used to identify *L. sacra*-specific mutations, and the effects of mutation were estimated using PROVEAN v1.1.5 [91], and a mutation would be classified as deleterious if its PROVEAN score was less than −2.5. We also attempt to detect convergent mutated sites between *L. sacra* and *T. baileyi*. To do this, a python script was used to screen all the sites of single-copy gene alignments to find mutation sites only occurring in these two species. The alignments of the selective genes were visualized with R package msa [92]. The domain architectures of selected PSGs and convergent genes were explored with SMART (https://smart.embl-heidelberg.de/, accessed on 16 April 2022) [93], and their tertiary structure was predicted with alphafold v2.0.0 in the casp14 model [94].

### 4.8. Chromosome Synteny with S. undulatus

The synteny between the *L. sacra* scaffold and *S. undulatus* chromosomes was conducted in JCVI v1.1.11 (https://github.com/tanghaibao/jcvi, accessed on 2 March 2022) utility libraries for python [95]. Firstly, orthologous proteins between the two species were confirmed with the jcvi.compara.catalog module, and block subsets were built based on the anchor file, which contained the genomic coordinates of each gene. The relationships of each block were visualized with the jcvi.graphics.karyotype module. 

### 4.9. Enrichment Analysis and Structural Prediction

GO and KEGG terms were obtained through the annotation pipelines described above. Similar to previous studies [96], R package clusterProfiler v4.0.1 was applied for GO enrichment analyses [97]. All annotated genes were set as background. A hypergeometric test was performed to obtain the *p*-value for each GO term, which was then corrected by the Benjamini–Hochberg false discovery rate (FDR) multiple-test. GO terms with a corrected *p*-value < 0.05 were considered significantly enriched. For KEGG enrichment, KO identifiers map were firstly reconstructed with the KEGG Mapper Reconstruct tool on website https://www.genome.jp/kegg, accessed on 24 June 2022. The KO number in each reconstructed pathway was parsed and counted using self-made python scripts. A hypergeometric test was performed using KO counts from both the foreground and background reconstructed pathways. KEGG terms with a *p*-value < 0.05 were considered significantly enriched.

## 5. Conclusions

Studying the genomes of Tibetan-endemic animals can provide a better understanding of the genetic mechanisms of highland adaptation. Based on our high-quality assembly of the *L. sacra* genome, together with genomic data from other high- and low-altitude species, we identified common molecular adaptations involved in angiogenesis, oxidative phosphorylation, adipocyte thermogenesis, and DNA repair among plateau animals. Genetic mechanisms related to the adaptation of ectotherms to their harsh environment were also explored, revealing potential lizard-specific adaptations as well as convergence between highland reptiles.

## Data Availability

The draft genome sequences and annotations, along with the raw reads for the paired-end and PacBio libraries, have been deposited in the National Genomics Data Center (NGDC) (https://ngdc.cncb.ac.cn, accessed on 2 August 2022) under the project accession code PRJCA010949.

## Supplementary Materials

The following supporting information can be downloaded at: https://www.mdpi.com/article/10.3390/ijms231710081/s1.
