# Supplementary material for: Genome of Laudakia sacra Provides New Insights into High-Altitude Adaptation of Ectotherms
Source: Int J Mol Sci. 2022 Sep 3;23(17):10081. doi: 10.3390/ijms231710081 (PMC9456099; doi:10.3390/ijms231710081)
Supplement: Supplementary file 1 [file ijms-23-10081-s001.zip › Lsac_manu.supple.prof.pdf]

Table S1. Sequencing data for *L. sacra* genome *de novo* assembly.

Platform	Category	Information	Sample ID
Illumina	Read Length (bp)	150	LJT_LAB2020323e
	Raw Paired-end Reads	982,090,054	
	Raw Bases (Gbp)	147.31	
	Clean Paired-end Reads	957,317,052	
	Clean Bases (Gbp)	143.6	
	Clean Bases Rate (%)	97.48	
	Q20 (%)	96.8	
	Q30 (%)	89.5	
	GC content (%)	41.9	
PacBio	Raw subreads reads	5,371,126	LJT_LAB2020323e
	Raw subreads base (Gbp)	163.03	
	Average raw subreads length	30,354	
	Raw subreads n50	49,223	
	Clean subreads reads	6,934,738	
	Clean subreads base (Gbp)	162.9	
	Average clean subreads length	23,491	
	Raw subreads n50	36,420	

Table S2. Assembly results of *L. sacra* genome.

	Contig length(bp)	Sequence number
Max length	77,011,479	
N10	60,031,939	3
N30	40,344,078	10
N50	20,185,579	23
N70	13,043,423	46
N90	4,912,859	89
Total length	1,797,983,225	284
GC content	0.427	

Table S3. Completeness of *L. sacra* genome estimated by BUSCO.

	Gene number	Proportion
Complete BUSCOs (C)	2419	93.5%
Complete and single-copy BUSCOs (S)	2382	92.1%
Complete and duplicated BUSCOs (D)	37	1.4%
Fragmented BUSCOs (F)	106	4.1%
Missing BUSCOs (M)	61	2.4%
Total BUSCO groups searched	2586	-

Table S4. Functional annotation of *L. sacra* genome from different databases.

Database	Gene number	Annotation ratio
KOG	13,428	67.50%
KEGG	13,273	66.72%
NR	19,155	96.29%
SwissProt	18,755	94.28%
GO	13,310	66.91%
Total cds sequence	19,893	-
Overall annotated	19,382	97.43%

Table S5. Repeat sequences annotation of *L. sacra* genome.

Class	Order	Superfamily	Number of elements	Length of sequence (bp)	Percentage of sequence (%)
	LINE		1164353	333902561	18.57
		RTE-X	67786	13992540	0.78
		L2	279585	89744560	4.99
		CR1	239676	93885168	5.22
		RTE-BovB	116751	33495916	1.86
		Unknown	239232	57199054	3.18
		L1	44386	14798038	0.82
		Penelope	107058	19504010	1.08
		Dong-R4	12266	3410845	0.19
		I-Jockey	20170	3686731	0.21
		Rex-Babar	9194	3011217	0.17
		Other	28249	1174482	0.07
Class I	SINE		371987	42776904	2.38
		tRNA-Deu	64244	11614412	0.65
		MIR	102895	13134780	0.73
		tRNA-RTE	34038	4435233	0.25
		Unknown	138528	11082448	0.62
		Other	32282	2510031	0.14
	LTR		600573	119444464	6.64
		Unknown	428288	65390301	3.64
		DIRS	19053	11515043	0.64
		ERVK	19408	2235430	0.12
		Ngaro	17441	21078963	1.17
		Gypsy	59954	11793157	0.66
		ERV1	34955	3114595	0.17
		Copia	11691	3835702	0.21

		Other	9783	481273	0.03
Class II	DNA		1119387	143491673	7.98
		Unknown	575007	70542126	3.92
		Maverick	36195	2374710	0.13
		CMC-EnSpm	47263	2494560	0.14
		hAT-Ac	89525	29511684	1.64
		TcMar-Tc1	22534	4561618	0.25
		hAT-Charlie	42673	6563774	0.37
		hAT-Tip100	57404	6173390	0.34
		hAT-Tag1	20464	3998552	0.22
		TcMar-Tigger	41640	6425147	0.36
		Other	186682	10846112	0.6
	MITE		147111	20873029	1.16
		Unknown	147111	20873029	1.16
	RC		19832	754904	0.04
		Other	19832	754904	0.04
Total TEs			3423243	661243535	36.78
Tandem Repeats			320316	9843940	0.55
	SSR		195589	2670989	0.15
	tandem repeat		124727	7172951	0.4
Unknown			511132	81796964	4.55
Simple repeats			39943	4637876	0.26
Other			26179	1908592	0.11
Low complexity			1633	269388	0.01
Total Repeats			4322446	759700295	42.25

Table S6. Genome information of species used in comparative genomic analysis.

Species	Abbreviation	Genome source
Thermophis baileyi	Tbai	GWHBJWY00000000 (https://ngdc.cncb.ac.cn/gwh)
Pseudonaja textilis	Ptex	GCA_900608585.1*
Pantherophis guttatus	Pgut*	GCF_001185365.1*
Protobothrops mucrosquamatus	Pmuc*	GCF_001527695.2*
Ophiophagus hannah	Ohan*	GCA_000516915.1*
Python bivittatus	Pbiv*	GCF_000186305.1*
Thamnophis sirtalis	Tsir*	GCF_001077635.1*
Crotalus viridis viridis	Cvir*	GCA_003400415.2*

Notechis scutatus	Nscu*	GCF_900518725.1*
Deinagkistrodon acutus	Dacu*	ftp.cngb.org/pub/gigadb/pub/10.5524/100001_101000/100196/
Thamnophis elegans	Tele*	GCF_009769535.1*
Naja naja	Nnaj	GCA_009733165.1*
Boa constrictor	Bcon	https://doi.org/10.6084/m9.figshare.9793013.v2
Hydrophis curtus	Hcur	https://doi.org/10.6084/m9.figshare.11391606.v5
Sceloporus undulatus	Sund*	GCF_019175285.1*
Paroedura picta	Ppic*	GCA_003118565.1*
Pogona vitticeps	Pvit*	GCF_900067755.1*
Shinisaurus crocodilurus	Scro*	https://www.ebi.ac.uk/ena/browser/view/GCA_021292165.1
Ophisaurus gracilis	Ogra*	http://gigadb.org/dataset/100119
Laudakia sacra	Lsac*	GWHBKHB00000000 (https://ngdc.cncb.ac.cn/gwh)
Lacerta viridis	Lvir*	GCA_900245905.1*
Gekko japonicus	Gjap*	GCF_001447785.1*
Anolis carolinensis	Acar*	GCF_000090745.1*
Lacerta agilis	Lagi*	GCF_009819535.1*
Podarcis muralis	Pmur*	GCF_004329235.1*
Zootoca vivipara	Zviv*	GCF_011800845.1*
Salvator merianae	Smer*	GCA_003586115.2*
Varanus komodoensis	Vkom*	GCF_004798865.1*
Chrysemys picta bellii	Cpic*	ftp.ensembl.org:/pub/release-102/fasta/chrysemys_picta_bellii
Pelodiscus sinensis	Psin*	ftp.ensembl.org:/pub/release-102/fasta/pelodiscus_sinensis
Chelonia mydas	Cmyd*	GCF_015237465.1*
Alligator mississippiensis	Amis*	GCF_000281125.3*
Crocodylus porosus	Cpor*	ftp.ensembl.org:/pub/release-102/fasta/crocodylus_porosus
Alligator sinensis	Asin*	GCF_000455745.1*
Latimeria chalumnae	Lcha	ftp.ensembl.org:/pub/release-102/fasta/latimeria_chalumnae
Nanorana parkeri	Npar	GCF_000935625.1*
Xenopus tropicalis	Xtro	ftp.ensembl.org:/pub/release-102/fasta/xenopus_tropicalis
Leptobrachium leishanense	Llei	GCA_009667805.1*
Mus musculus	Mmus	ftp.ensembl.org:/pub/release-102/fasta/mus_musculus
Homo sapiens	Hsap	ftp.ensembl.org:/pub/release-102/fasta/homo_sapiens
Gallus gallus	Ggal*	ftp.ensembl.org:/pub/release-102/fasta/gallus_gallus

Anas platyrhynchos	Apla	ftp.ensembl.org:/pub/release-102/fasta/anas_platyrhynchos
Parus humilis	Phum	GCF_000331425.1*
Bos grunniens	Bgru	GCF_000298355.1*
Ochotona curzoniae	Ocur	GCF_017591425.1*
Equus caballus	Ecab	GCF_002863925.1*
Falco naumanni	Fnau	GCF_017639655.2*
Ornithorhynchus anatinus	Oana	GCF_004115215.2*
Parus major	Pmaj	GCF_001522545.3*
Taeniopygia guttata	Tgut	GCF_003957565.2*

Note: Abbreviation names with * marked genomes used for PSGs and QEGs analysis; Genome source with * denote assembly accession Number in the National Center for Biotechnology Information.

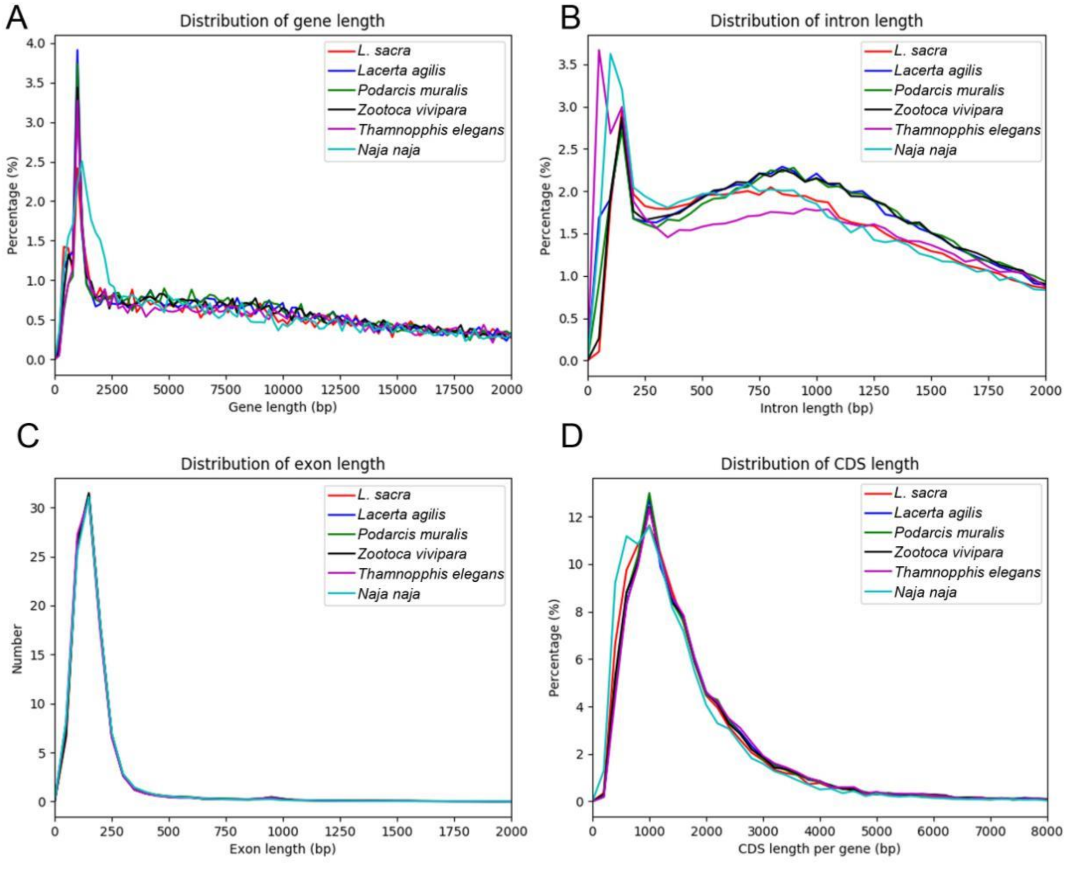

Figure S1. Comparison of length distribution of annotated genes (A), introns (B), exons (C) and CDS (D) between *L. sacra* and other five species.

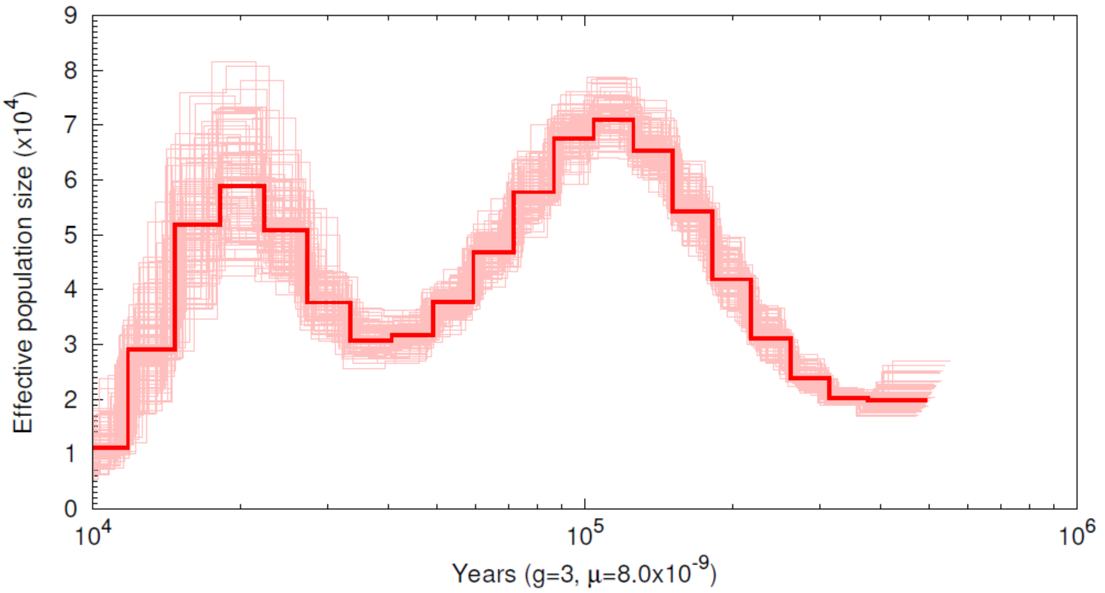

Figure S2. Changes in effective population size (*Ne*) of *L. sacra* through time inferred by the Pairwise Sequentially Markovian Coalescent model. The generation time (g) was set to 3 years and a mutation rate (μ) was 8×10^{-9} per site per generation.

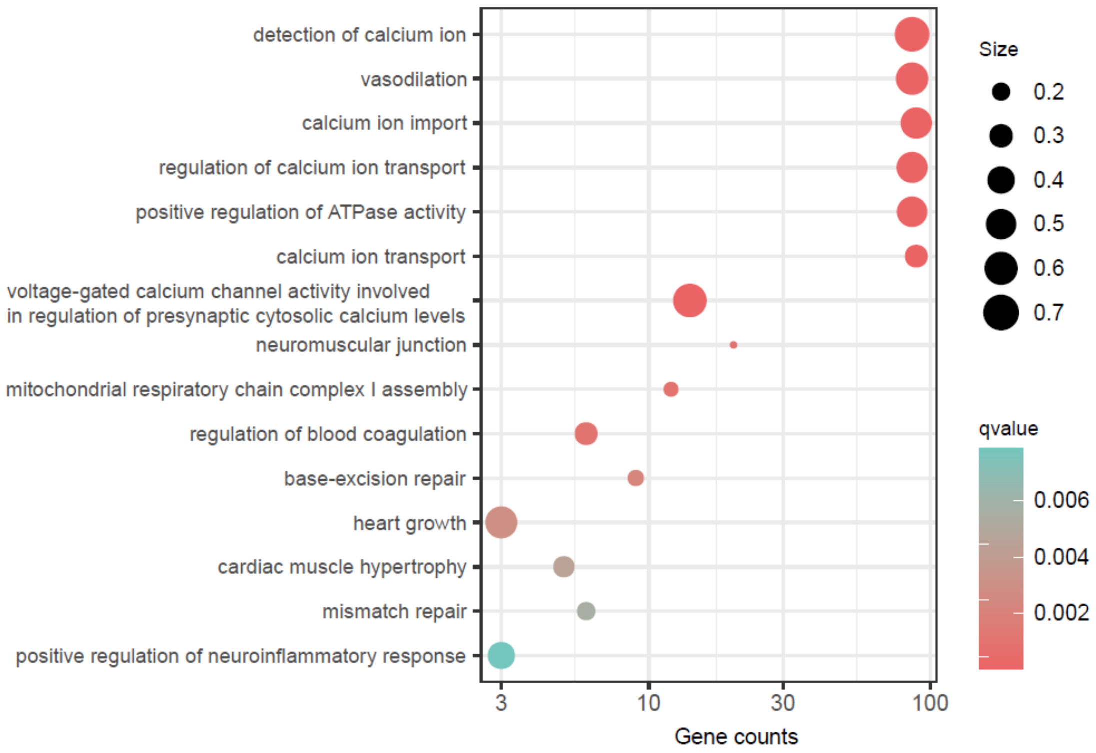

Figure S3. GO enrichment result of genes from significantly expanded gene families of *L. sacra*.

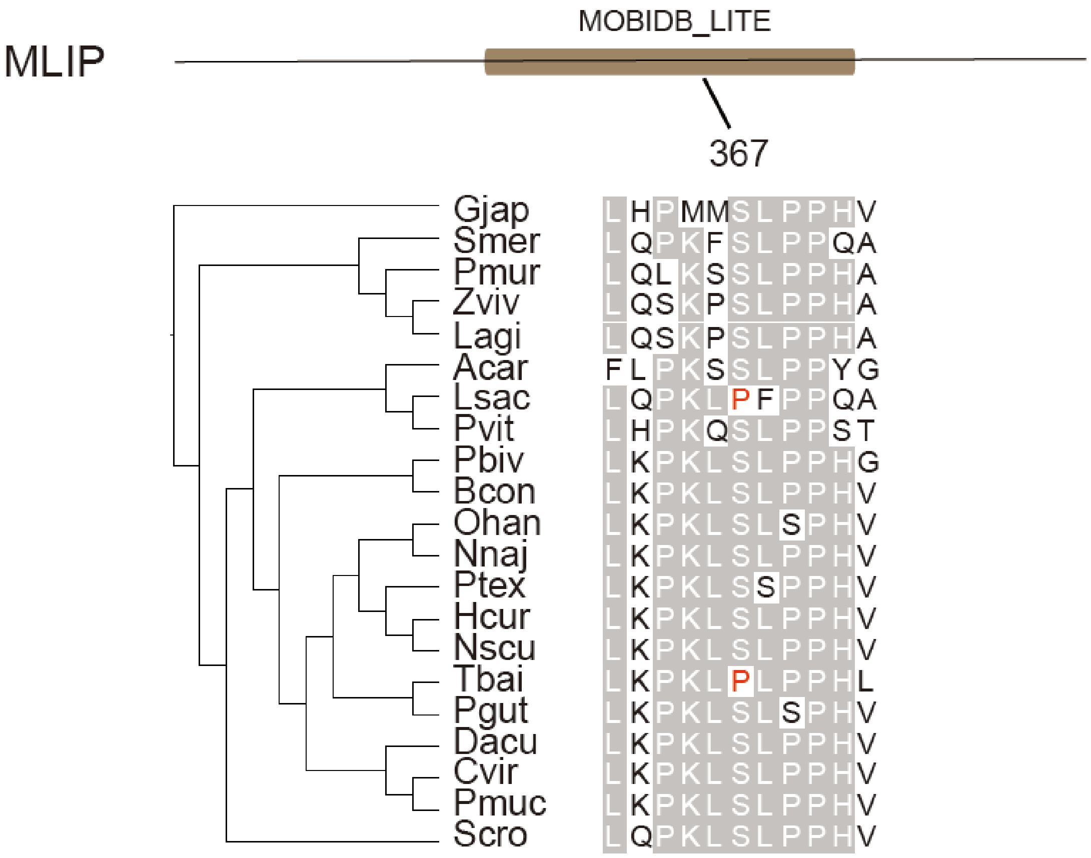

Figure S4. Sequence alignment of MLIP. MLIP has a S367P convergent amino acid replacement in *L. sacra* and *T. baileyi*, which is conserved among low-land reptiles.
